# Supplementary material for: Engineering a synthetic gene circuit for high-performance inducible expression in mammalian systems
Source: Nat Commun. 2024 Apr 17;15:3311. doi: 10.1038/s41467-024-47592-y (PMC11024104; doi:10.1038/s41467-024-47592-y)
Supplement: Supplementary file 1 — Supplementary Information [file 41467_2024_47592_MOESM1_ESM.pdf]

Supplementary Information for:

Engineering a Synthetic Gene Circuit for High-Performance Inducible Expression in Mammalian Systems

AUTHORS

Giuliano De Carluccio [1,2], Virginia Fusco [1,2], Diego di Bernardo[1,2,*].

AFFILIATIONS

[1]Telethon Institute of Genetics and Medicine, Naples, Italy

[2]University of Naples Federico II, Department of Chemical Materials and Industrial Engineering, Naples, Italy

*Correspondence should be addressed to D. di Bernardo: dibernardo@tigem.it

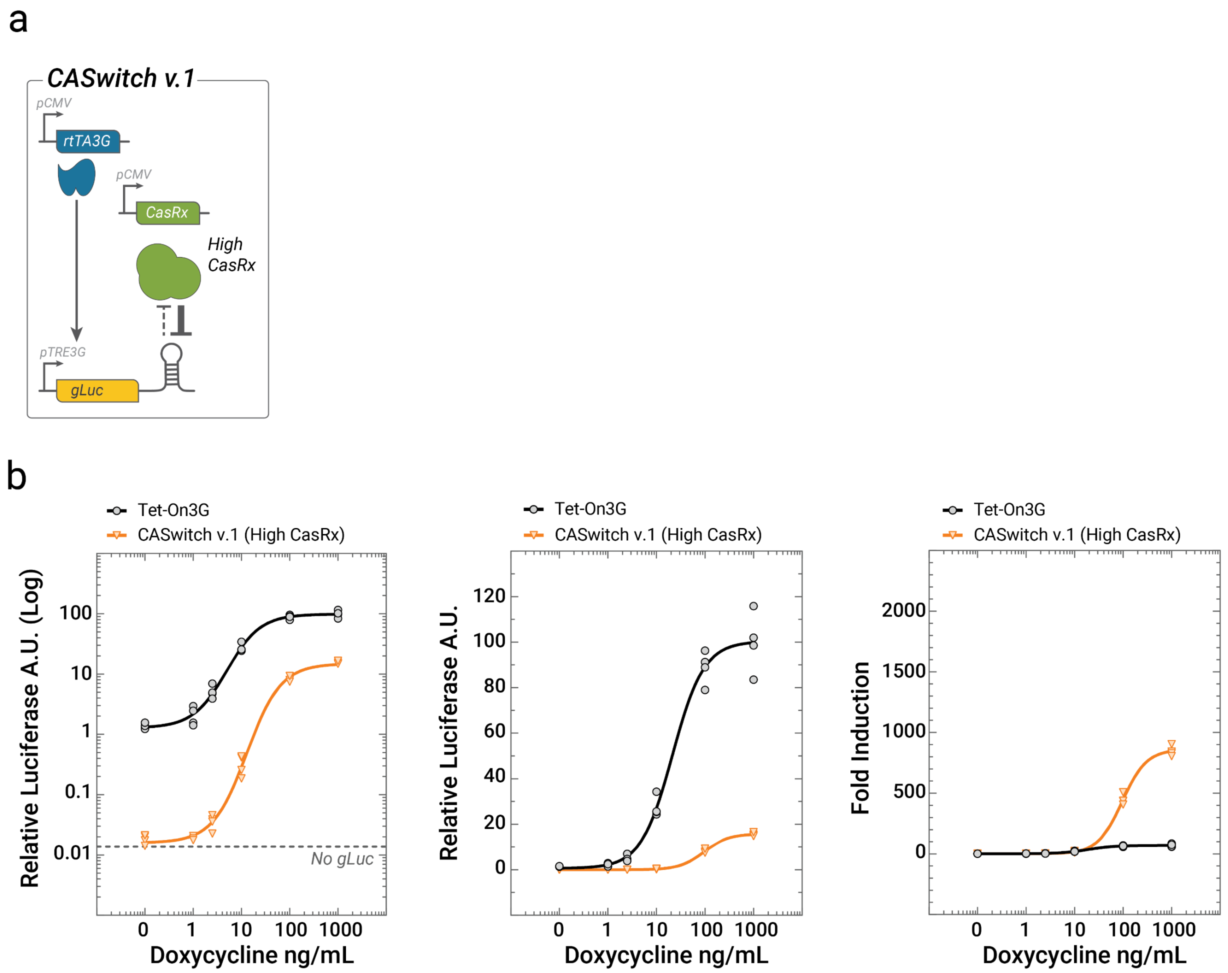

Supplementary Figure 1. Characterization of the CASwitch v.1 system at higher relative concentrations of the CasRx endoribonuclease. (a) CASwitch v.1 inducible gene expression system. The rtTA3G and the CasRx are constitutively expressed from constitutive pCMV promoter, while the gLuc with the Direct Repeat (DR) is placed downstream of the pTRE3G promoter. Cells were transfected with a molar ratio between pCMV-rtTA3G : pTRE3G-gLuc-DR : pCMV-CasRx plasmids of 1:5:5 **(b)** Experimental validation of CASwitch v.1 at relative higher concentration of CasRx (High CasRx, orange triangles) and comparison with the state-of-the-art Tet-On3G gene expression system (black circles) at the indicated concentrations of doxycycline. n=4 biological replicates are shown. Relative Luciferase A.U. of each data point is shown as the percentage of the mean of Luciferase A.U. value of the Tet-On3G system at 1000 ng/mL of doxycycline and plotted in a log-scale, linear-scale, and in as fold-induction values computed as the ratio between Relative Luciferase A.U of each data point and the mean of Relative Luciferase A.U in the absence of doxycycline. "No gLuc" represents the average Luciferase A.U. values of n=4 biological replicates of cells that were not transfected with the pTRE3G-gLuc-DR construct but only the pCMV-RedFireFly plasmid as transfection control and shows the instrument background signal.

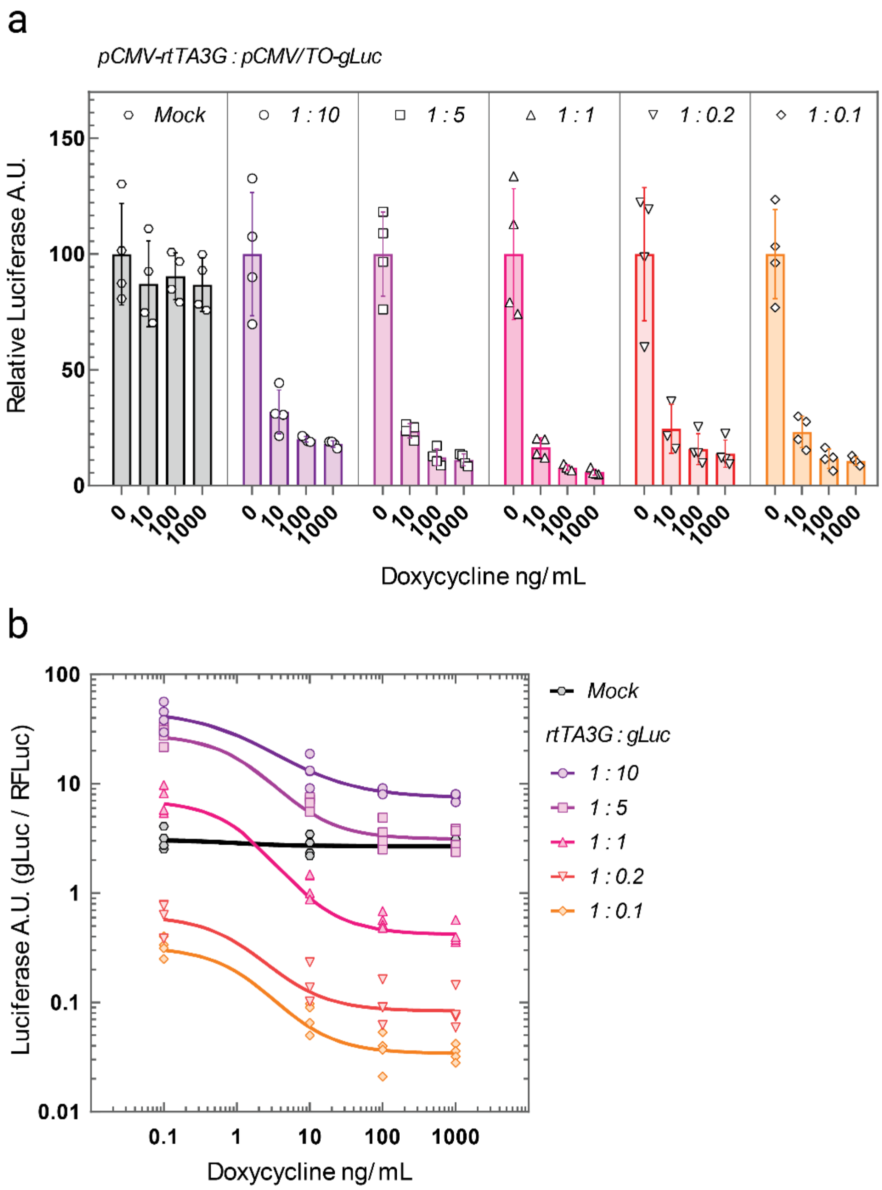

Supplementary Figure 2. pCMV/TO promoter characterization. Experimental validation of pCMV/TO transcriptional inhibitory function. HEK293T cells are transfected with two plasmids, one for the rtTA3G inducible transcriptional factor construct and the other the gLuc construct driven by the pCMV/TO promoter at the indicated relative concentrations. Luciferase expression was evaluated by dual-luminescence measurements at increasing amount of doxycycline. (a) Luciferase expression is plotted as Relative Luciferase A.U. which is the ratio between the Luciferase A.U. value of each data point and the mean of Luciferase A.U. values of the pCMV/TO-gLuc in the absence of doxycycline. Error bars correspond to the standard deviation for n = 4 biological replicates (b) Luciferase A.U. values calculated as the ratio between the luminescence value of gLuc and Red Firefly Luciferase of each data point.

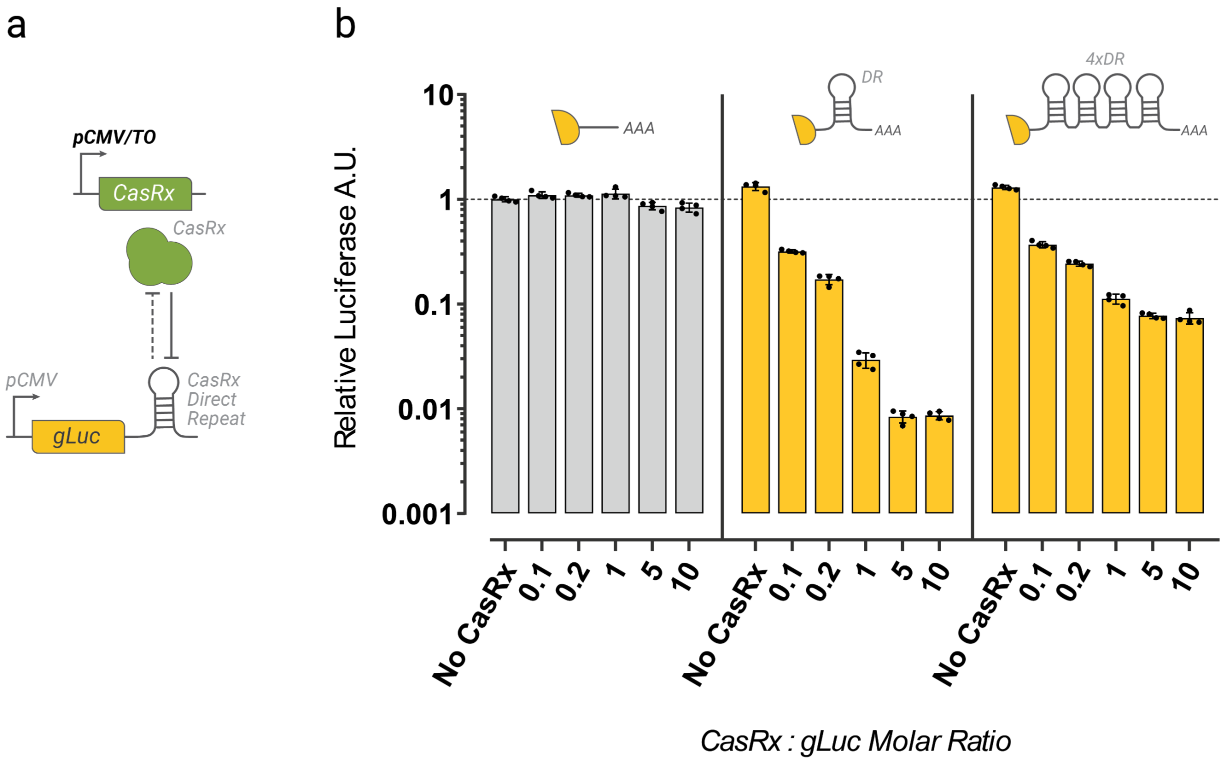

Supplementary Figure 3. Mutual inhibition module featuring pCMV/TO-CasRx (a) Schematic of the experimental implementation of the mutual inhibition module featuring a pCMV/TO-driven CasRx. Cells are transfected with two plasmids at the indicated relative concentrations, one for the pCMV/TO CasRx construct and the other for the gLuc construct harboring no, one and four repetitions of the Direct Repeat (DR) in the 3'Untranslated Region (3'UTR) of the gLuc reporter gene. **(b)** Experimental verification of the modified MI module. gLuc expression was evaluated by dual luminescence measurements resulting in the Luciferase A.U. value, calculated as the ratio between gLuc and Red Firefly Luciferase luminescence for each data point. Results are plotted as Relative Luciferase A.U. representing the ratio between the Luciferase A.U. value of each data point and the mean of Luciferase A.U. values of the gLuc without DR in the absence of any CasRx. Error bars correspond to the standard deviation for n = 4 biological replicates.

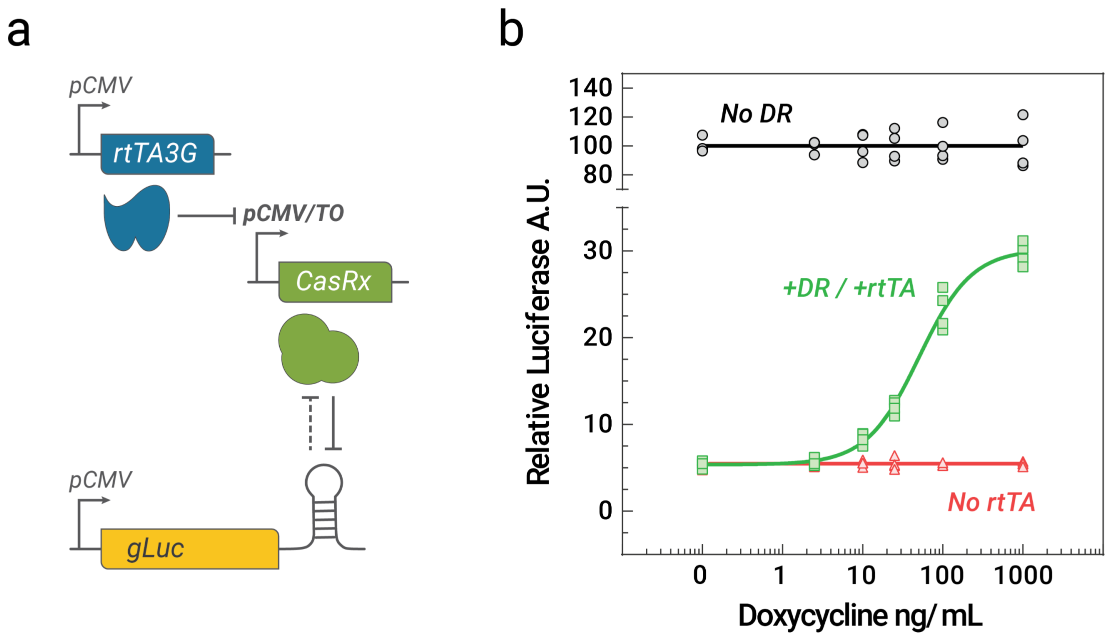

Supplementary Figure 4. pCMV/TO-mediated repression of CasRx inhibitory function. (a) Schematics of the experimental implementation testing pCMV/TO-mediated repression of CasRx. In the presence of doxycycline rtTA3G attaches to binding sites downstream the pCMV/TO TATA site, causing transcriptional repression of the CasRx, that in turn should results in increasing gLuc expression **(b)** Experimental validation at the indicated concentrations of doxycycline. gLuc expression was evaluated by dual luminescence measurements resulting in the Luciferase A.U. value calculated as the ratio between gLuc and Red Firefly Luciferase luminescence for each data point. Results are plotted as Relative Luciferase A.U. representing the ratio between the Luciferase A.U. value of each data point and the mean of Luciferase A.U. values of the gLuc with DR in the absence of doxycycline. n=4 biological replicates are shown. No DR (black), cells are transfected with a gLuc construct harboring no DR in the 3'UTR; +DR/+rtTA (green), cells are transfected with three plasmid encoding rtTA3G, pCMV/TO driven CasRx, and a gLuc featuring a DR in its 3'UTR; No rtTA (red), cell are not transfected with rtTA3G.

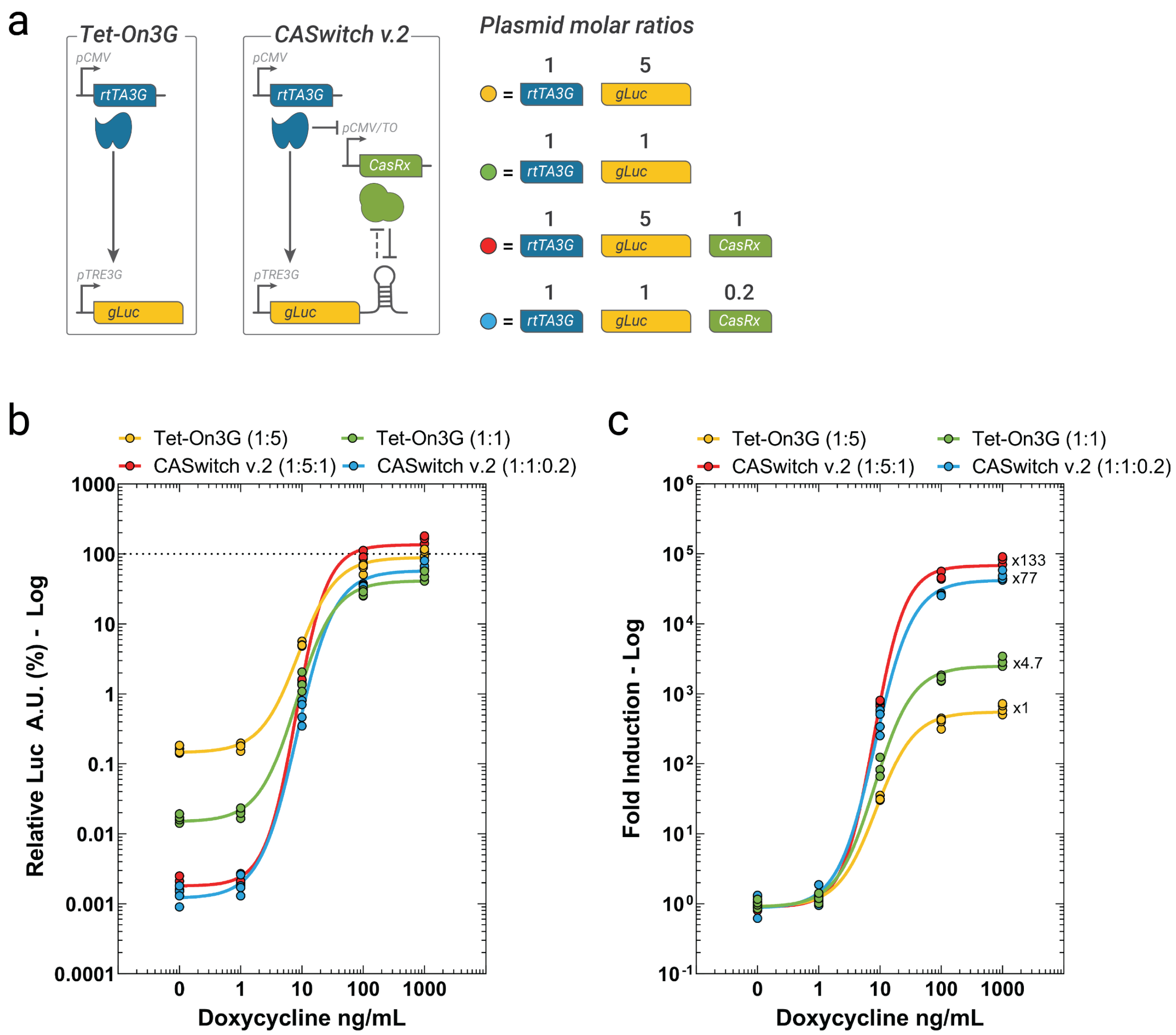

Supplementary Figure 5. Comparison of the CASwitch v.2 and Tet-On3G systems at different plasmids molar ratios. (a) The two plasmids encoding the Tet-ON3G system (pCMV-rtTA3G:pTRE3G-fLuc-DR) and the three plasmids encoding the CASwitch v.2 system (pCMV-rtTA3G : pTRE3G-fLuc-DR : pCMV/TO-CasRx) were transfected at the indicated molar ratios in HEK393T cells; **(b)** Relative Luciferase Arbitrary Units A.U. in logarithmic scale computed as the percentage of the mean of Luciferase A.U. value of the Tet-On3G (1:5) system at 1000 ng/mL of doxycycline for the indicated systems and plasmid ratios; **(c)** Fold Induction in logarithmic scale computed as the ratio between relative luciferase A.U of each data point and the mean of relative luciferase A.U in the absence of doxycycline. The x symbol indicates how many times the maximal fold induction increases over the one of the standard Tet-On3G system at a plasmid ratio of (1:5) used as a reference. n=3 biological replicates per point.

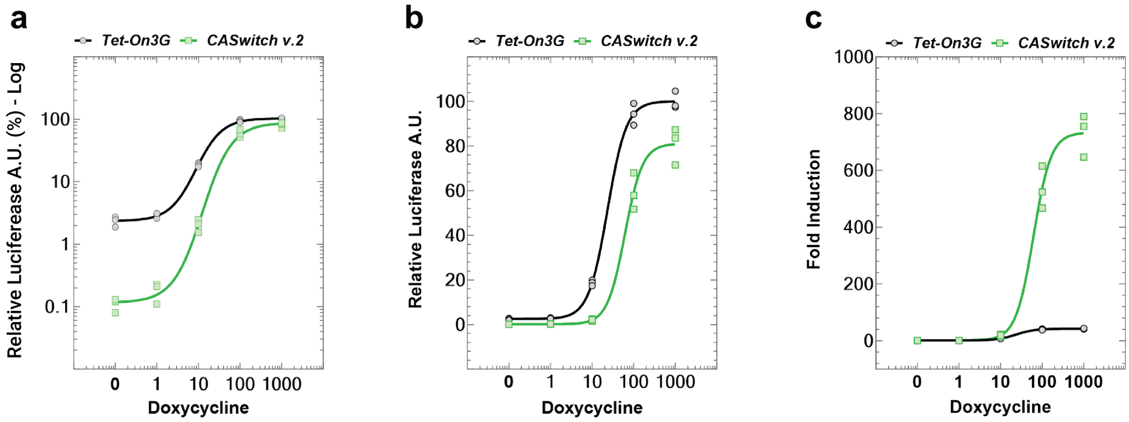

Supplementary Figure 6. Characterization of the CASwitch v.2 performance in HeLa cells. Experimental validation of CASwitch v.2 (green) and comparison with the state-of-the-art Tet-On3G gene expression system (black) at the indicated concentrations of doxycycline. **(a,b)** Relative Luciferase A.U. computed as the percentage of the mean Luciferase A.U. value of the Tet-On3G system at 1000 ng/mL of doxycycline and shown either in logarithmic scale (a) or in linear scale (b); **(c)** Fold induction values calculated as the ratio between Relative Luciferase A.U (%) of each data point and the mean of Relative Luciferase A.U in the absence of doxycycline. n=3 biological replicates per point.

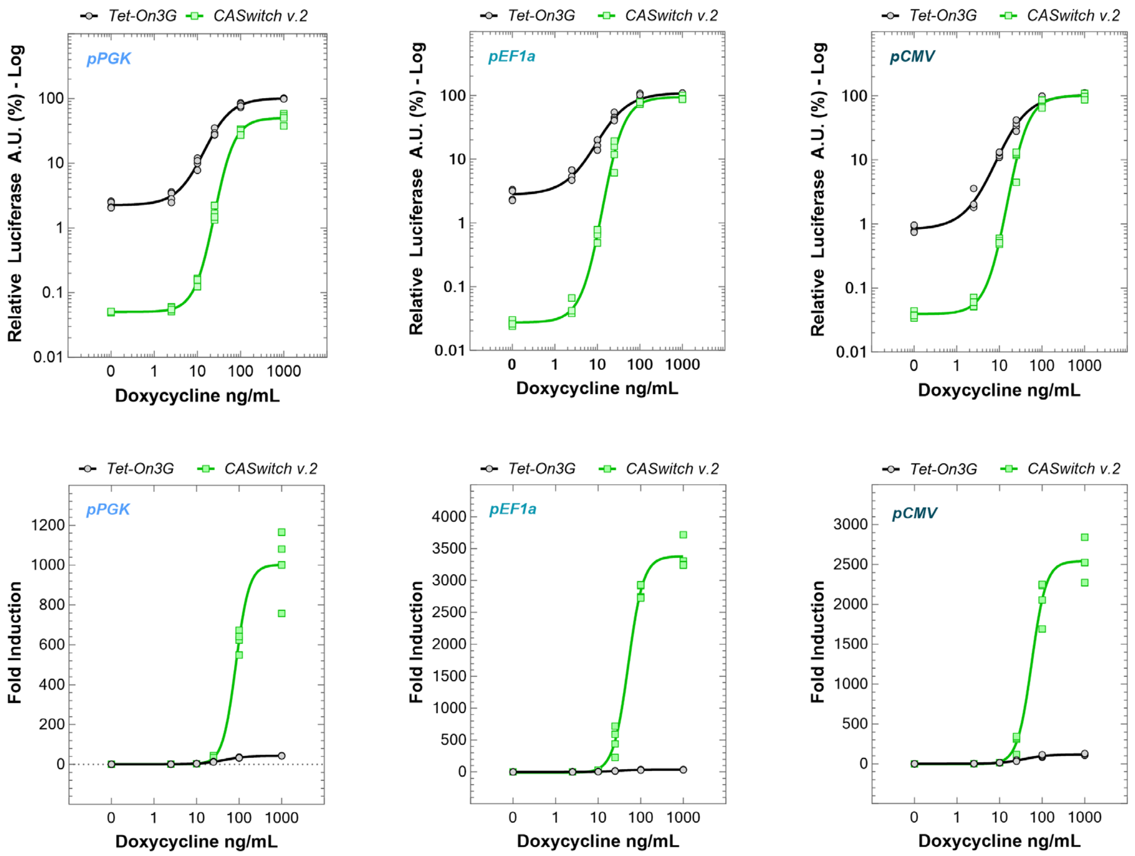

Supplementary Figure 7. Characterization of the CASwitch v.2 performance varying the expression strength of the promoter driving the rtTA3G. (a) Schematics of the CASwitch v.2 system featuring promoters with decreasing expression strengths: pCMV, pEF1a, pPGK. **(b)** Experimental validation of CASwitch v.2 variants (green lines, promoter used indicated up left) and comparison with the state-of-the-art Tet-On3G gene expression system (black) at the indicated concentrations of doxycycline. n=4 biological replicates are shown. Relative Luciferase A.U., shown in logarithmic scale, is calculated as the percentage of the mean of Luciferase A.U. value of the Tet-On3G system at 1000 ng/mL of doxycycline. **(c)** Fold-induction is computed as the ratio between Relative Luciferase A.U of each data point and the mean of Relative Luciferase A.U in the absence of doxycycline.

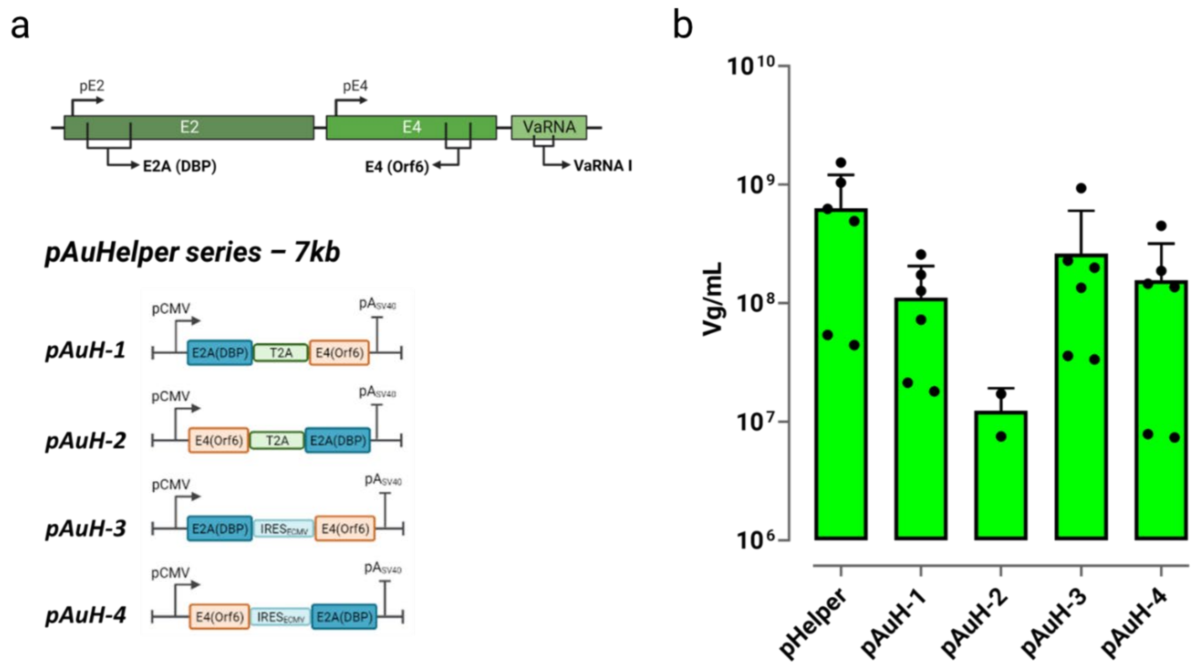

Supplementary Figure 8. a) Schematic of the Human Adenovirus-5 (HAdV-5) genes found in common Helper plasmids used for AAV vector manufacturing. pAμHelper plasmids schematic. E2A(DBP) and E4(Orf6) coding sequences were taken from the wt HAdV-5 genes to design a single transcriptional unit by means of the EMCV-IRES or P2A-skipping ribosome sequence. By exchanging the positions of E2A(DBP) and E4(Orf6) in the bicistronic transcriptional units, we generated four different Adenovirus Helper plasmids. **b)** AAV production yield quantification by means of qPCR. Cells were transfected with pTransgene, pPackaging, and pHelper or one of the pAμHelper plasmids. Vg/mL represents the viral genome (Vg) concentration used to quantify the production yields obtained using the indicated Helper plasmid. The error bars represent the mean and standard deviation of replicates within three independent experiments (n = 6), albeit for pAuH-2 where n = 2.

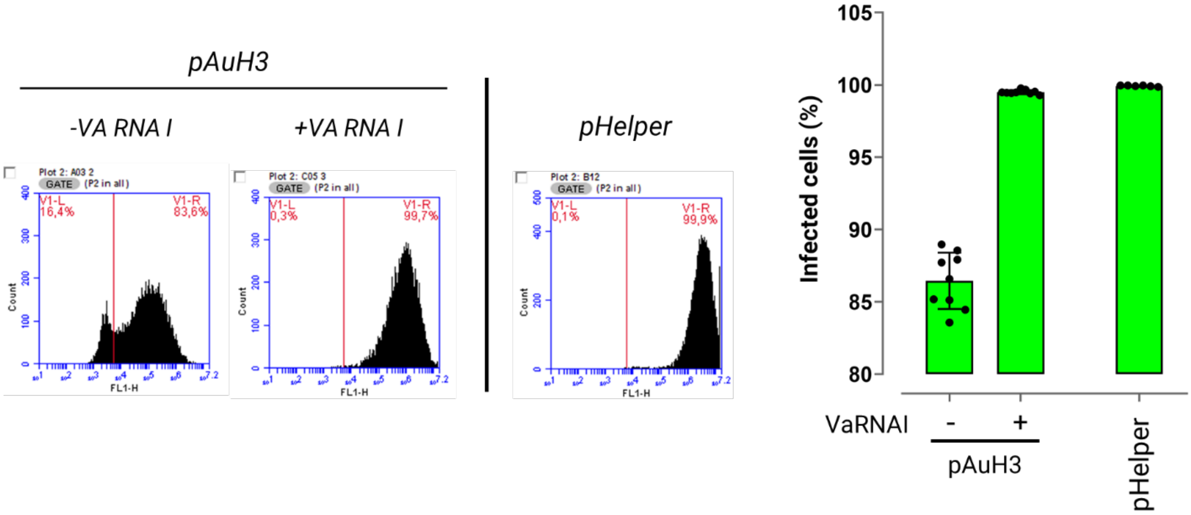

Supplementary Figure 9. VaRNA-I fully restore production capacity for AµH-3 Helper plasmid. a) Flow cytometry analyses of cells transduced with cell lysates containing AAV vector produced using the pAµH-3 co-transfected either with or not VaRNA-I expressing plasmid and using the standard pHelper plasmid. Representative flow cytmoterycytometry histograms (left) and their quantification (right) are also shown. The error bars represent the mean and standard deviation of n = 9 biological replicates. The percentage of transduced cells is calculated as the percentage of GFP+ cells, setting the cell autofluorescence threshold to that of non untransduced cells. At least 10,000 cells were analyzed for each point.

Supplementary Notes for:

Engineering a Synthetic Gene Circuit for High-Performance Inducible Expression in Mammalian Systems

AUTHORS

Giuliano De Carluccio [1,2], Virginia Fusco [1,2], Diego di Bernardo[1,2,*].

AFFILIATIONS

[1]Telethon Institute of Genetics and Medicine, Naples, Italy

[2]University of Naples Federico II, Department of Chemical Materials and Industrial Engineering, Naples, Italy

*Correspondence should be addressed to D. di Bernardo: dibernardo@tigem.it

Mathematical modelling of synthetic circuits for high-performance inducible gene expression systems.

We set to model the four circuits reported in Figure 1a of the main manuscript, i.e. the naive inducible gene expression system (TF), the coherent feed forwad type IV (CFFL4), the Mutual Inhibition (MI) an the Coherent Inhibitory Loop (CIL). All the models consist of a set of pseudo-chemical reaction equations useful to capture changes in the concentrations of the main biochemical species in the systems: the transcriptional activator X; the reporter Z; the repressor Y; and the inducible molecule D. The equations are derived through the law of mass-action and Michaelis-Menten kinetics by assuming that: (i) the transcription dynamics are at a quasi-steady-state; (ii) the production rates of constitutive promoters are constant over time; (iii) the production rates of inducible promoters can be described using Hill functions, where α_i^0, α_i, κ_i and n_i denote the basal transcription rate, the maximum induced transcription rate, the activation coefficient, and the Hill coefficient of the species i, respectively; (iv) the degradation kinetic is of first-order with rate γ_i; (v) the inducer molecule concentration can be modified over time; (vi) the sequestration between species i and j is modeled with first-order kinetic with rate κ_{ij}. For any variable, e.g. X, we denote its steady-state values as $\bar{X}$, for some non-zero D value, and with $\bar{X}^0$ when $D = 0$.

Model of the TF synthetic circuit topology

In the model, protein X is constitutively expressed (i.e. produced at a constant rate) and it binds its cognate promoter only in the presence of molecule D, thus activating the reporter gene Z:

$$\frac{dX}{dt} = u_1 - \gamma_X X, \tag{1}$$

$$\frac{dZ}{dt} = a_Z(X) - \gamma_Z Z, \tag{2}$$

where:

$$a_Z(X) := \alpha_Z^0 + \alpha_Z \frac{(DX)^{n_Z}}{{\kappa_Z}^{n_Z} + (DX)^{n_Z}}, \qquad \text{and} \qquad \bar{a}_i = a_i(\bar{X}). \tag{3}$$

The TF system has one equilibrium point:

$$\bar{X} = \frac{u_1}{\gamma_X}, \tag{4}$$

$$\bar{Z} = \frac{\bar{a}_Z}{\gamma_Z}. \tag{5}$$

The equilibrium point is locally stable, as the eigenvalues of the Jacobian are always negative real numbers:

$$\lambda_1 = -\gamma_X, \qquad \lambda_2 = -\gamma_Z. \tag{6}$$

Model of the CFFL4 synthetic circuit topology

In the model, protein X is constitutively expressed and, in the presence of molecule D, it binds the cognate promoter upstream of species Z, thus activating it, but it also binds a second promoter upstream of species

Y, thereby inhibiting its production. Species Y is modelled as a repressor able to inhibit production of the reporter species Z (for a full mathematical derivation refer to APPENDIX A):

$$\frac{dX}{dt} = u_1 - \gamma_X X, \tag{7}$$

$$\frac{dZ}{dt} = \frac{a_Z(X)}{1 + k_{zy}Y} - \gamma_Z Z, \tag{8}$$

$$\frac{dY}{dt} = b_Y(X) - \gamma_Y Y, \tag{9}$$

where:

$$b_Y(X) := \alpha_Y^0 + \alpha_Y \frac{{\kappa_Y}^{n_Y}}{(DX)^{n_Y} + {\kappa_Y}^{n_Y}}, \qquad \text{and} \qquad \bar{b}_i = b_i(\bar{X}). \tag{10}$$

The CFFL4 system has a single equilibrium point:

$$\bar{X} = \frac{u_1}{\gamma_X}, \tag{11}$$

$$\bar{Y} = \frac{\bar{b}_Y}{\gamma_Y}, \tag{12}$$

$$\bar{Z} = \frac{\bar{a}_Z}{(1 + k_{zy}\bar{Y})\gamma_Z}. \tag{13}$$

By assessing the eigenvalues of the Jacobian matrix of the system:

$$\lambda_1 = -\gamma_X, \qquad \lambda_2 = -\gamma_Z, \qquad \lambda_3 = -\gamma_Y, \tag{14}$$

we can deduce that the equilibrium point is locally stable.

Model of the MI synthetic circuit topology

In the model, protein X is constitutively expressed and, in the presence of molecule D, it binds the cognate promoter upstream of species Z and activates its production. Additionally, a repressor species Y is also constitutively expressed and enables a mutual inhibition regulatory interaction with species Z: specifically, species Y inhibits production of species Z as in the CFFL4 model, but is so doing it gets sequestered by stoichiometric interaction with species Z (for a full mathematical derivation refer to APPENDIX A):

$$\frac{dX}{dt} = u_1 - \gamma_X X, \tag{15}$$

$$\frac{dZ}{dt} = \frac{a_Z(X)}{1 + k_{zy}Y} - \gamma_Z Z, \tag{16}$$

$$\frac{dY}{dt} = u_2 - \gamma_Y Y - kY \frac{a_Z(X)}{1 + k_{zy} Y}. \tag{17}$$

The MI model has two equilibrium points with opposite signs, hence we consider only the positive one as the state variables represent concentrations. At the positive equilibrium point the values are:

$$\bar{X} = \frac{u_1}{\gamma_X}, \tag{18}$$

$$\bar{Y} = \frac{\sqrt{(\bar{a}_Z k + \gamma_Y - k_{zy} u_2)^2 + 4\gamma_Y k_{zy} u_2} - \bar{a}_Z)k - \gamma_Y + k_{zy} u_2}{2\gamma_Y k_{zy}}, \tag{19}$$

$$\bar{Z} = \frac{\bar{a}_Z}{(1 + k_{zy}\bar{Y})\gamma_Z}. \tag{20}$$

The eigenvalues of the Jacobian matrix of the system at this equilibrium point are:

$$\lambda_1 = -\gamma_X, \qquad \lambda_2 = -\gamma_Z, \qquad \lambda_3 = -\gamma_Y - \frac{\bar{a}_Z k}{(1 + \bar{Y} k_{zy})^2}, \tag{21}$$

therefore, the equilibrium point is locally stable.

Model of the CIL synthetic circuit topology

The CIL is a modification of the incoherent feedforward loop of type four (CFFL4) where repressor Y and species Z mutual inhibit each other. In the model, protein X is constitutively expressed and, in the presence of molecule D, it binds the cognate promoter upstream of species Z and activates its production, but it also binds a second promoter upstream of species Y, thereby inhibiting its production, as in the CFFL4 for model. In this case, however, species Y enables a mutual inhibition regulatory interaction with species Z, as in the MI model. The equations describing the CIL model are:

$$\frac{dX}{dt} = u_1 - \gamma_X X, \tag{22}$$

$$\frac{dZ}{dt} = \frac{a_Z(X)}{1 + k_{zy} Y} - \gamma_Z Z, \tag{23}$$

$$\frac{dY}{dt} = b_Y(X) - kY \frac{a_Z(X)}{1 + k_{zy} Y} - \gamma_Y Y. \tag{24}$$

The CIL system displays two equilibrium points with opposite signs; hence we focus on the positive one, as the species in question represent concentrations:

$$\bar{X} = \frac{u_1}{\gamma_X}, \tag{25}$$

$$\bar{Y} = \frac{\sqrt{(\bar{a}_Z k + \gamma_Y - k_{zy}\bar{b}_Y)^2 + 4\gamma_Y k_{zy}\bar{b}_Y} - \bar{a}_Z k - \gamma_Y + k_{zy}\bar{b}_Y}{2\gamma_Y k_{zy}}, \tag{26}$$

$$\bar{Z} = \frac{\bar{a}_Z}{(1 + k_{zy}\bar{Y})\gamma_Z}. \tag{27}$$

The eigenvalues of the Jacobian of the system at this equilibrium point are always negative thus it is locally stable:

$$\lambda_1 = -\gamma_X, \qquad \lambda_2 = -\gamma_Z, \qquad \lambda_3 = -\gamma_Y - \frac{\bar{a}_Z k}{(1 + \bar{Y}k_{yz})^2}. \tag{28}$$

Derivation of Fold Change Activation

In inducible gene expression systems, Fold-change activation (FCA) is defined as the ratio between the maximal achievable concentration of reporter protein in the presence of the inducer molecule, divided by its minimal concentration in the absence of stimulus (i.e. leakiness). We set to derive the FCA of the four models described above, i.e. TF, CFFL4, MI and CIL, by assessing and contrasting their fold-change expressions through both analytical and numerical methods.

The analytical FCA can be obtained from the locally stable equilibria of the four models:

$$FC_i = \frac{\bar{Z}_i(D)}{\bar{Z}_i(0)}, \tag{29}$$

where i is anyone of the four models, D stands for the concentration of the inducer molecule and Z is the reporter gene. Hence, for each of the four models, the FCA is the following:

$$FC_{TF} = \frac{\bar{a}_Z}{\bar{a}_Z^0}, \tag{30}$$

$$FC_{CFFL4} = \frac{\bar{a}_Z(\theta_Y + \bar{b}_Y^0)}{\bar{a}_Z^0(\theta_Y + \bar{b}_Y)}, \tag{31}$$

$$FC_{MI} = \frac{\bar{a}_Z\phi_Y - \theta_Y - u_2 + \sqrt{(\bar{a}_Z\phi_Y + \theta_Y - u_2)^2 + 4\theta_Y u_2}}{\bar{a}_Z^0\phi_Y - \theta_Y - u_2 + \sqrt{(\bar{a}_Z^0\phi_Y + \theta_Y - u_2)^2 + 4\theta_Y u_2}}, \tag{32}$$

$$FC_{CIL} = \frac{\bar{a}_Z\phi_Y - \theta_Y - \bar{b}_Y + \sqrt{(\bar{a}_Z\phi_Y + \theta_Y - \bar{b}_Y)^2 + 4\theta_Y \bar{b}_Y}}{\bar{a}_Z^0\phi_Y - \theta_Y - \bar{b}_Y^0 + \sqrt{(\bar{a}_Z^0\phi_Y + \theta_Y - \bar{b}_Y^0)^2 + 4\theta_Y \bar{b}_Y^0}}, \tag{33}$$

where:

$$\theta_y = \frac{\gamma_Y}{k_{zy}}, \tag{34}$$

$$\phi_Y = \frac{k}{k_{zy}}. \tag{35}$$

It is then easy to verify that the fold-changes of the synthetic circuits CFFL4, MI and CIL can be written as in terms of the fold-change of the TF model (FC_{TF}):

$$FC_{CFFL4} = FC_{TF}\frac{\theta_Y + \bar{b}_Y^0}{\theta_Y + \bar{b}_Y}, \tag{36}$$

$$FC_{MI} = FC_{TF}\frac{\theta_Y + \bar{Y}_{MI}^0}{\theta_Y + \bar{Y}_{MI}}, \tag{37}$$

$$FC_{CIL} = FC_{TF}\frac{\theta_Y + \bar{Y}_{CIL}^0}{\theta_Y + \bar{Y}_{CIL}}. \tag{38}$$

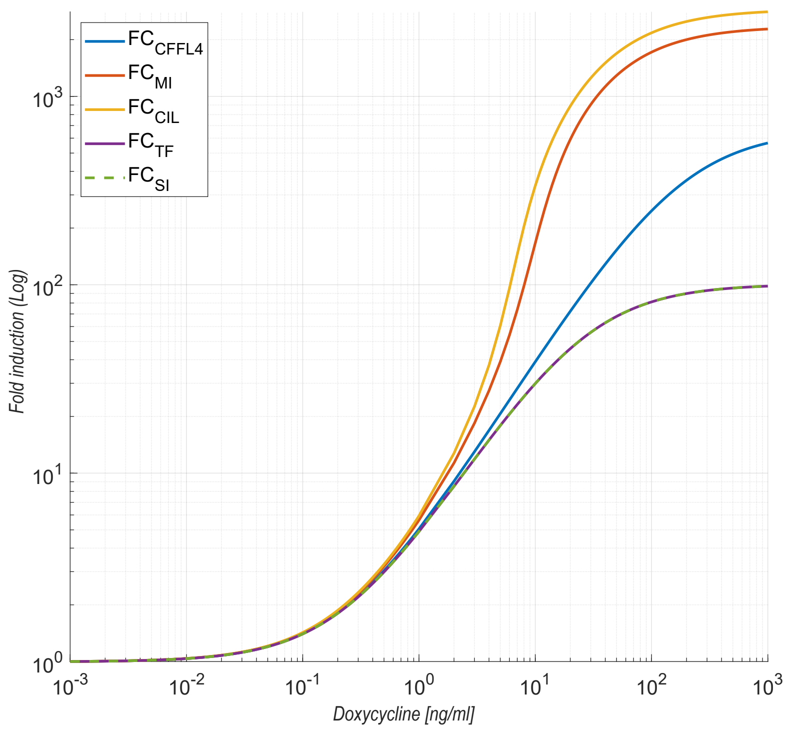

Figure 1: FCA of the models as a function of the inducer molecule. TF (1)-(2) (purple line), CFFL4 (7)-(8) (yellow line), MI (15)-(16) (blu line), CIL (22)-(23) (red line).

Given that the system parameters are real and positive, and that the functions in Eqs.(3) and (10) are respectively increasing and decreasing in the presence of drug D, then it can be shown that the quantities multiplying the FC_{TF} in each expression are always greater than one, implying that all the three circuits improve the FCA over the naive TF gene expression system, independently of the parameters' values.

To proceed in investigating the comparison of these three systems, we performed numerical simulations using the parameter values listed in Table 1, which were derived by fitting of the experimental data reported in the Figure 2 of the main manuscript. Results are reported in Figures 1, where it appears that the CIL is the best performing circuit in terms of FCA, closely followed by the MI circuit, whereas the CFFL4 has a poorer performance, even if it still improves over the naive TF topology. These results demonstrate that the mutual inhibition is a key feature to achieve high FCA.

To further explore the robustness of these results, we repeated numerical simulations by varying the parameters' values modeling the repression exerted by Y over Z, i.e. the rates of production of the Y promoters (i.e. α_Y and u_2), as well the strength of interaction between Y and Z (k_{zy}). Results are reported in Figures 2 and 3.

Simulation results confirm the previous findings, where the CFFL4 system performs less favorably compared to the MI and CIL systems. Specifically, the CFFL4 system exhibits lower output intensity compared to the other two systems, with CIL demonstrating the highest signal strength. Furthermore, both MI and CIL systems display significant improvements in fold-change activation, with CIL being the most effective.

Based on the analysis results, it is not advisable to implement the CFFL4 system biologically, as it exhibits inferior performance compared to the MI and CIL.

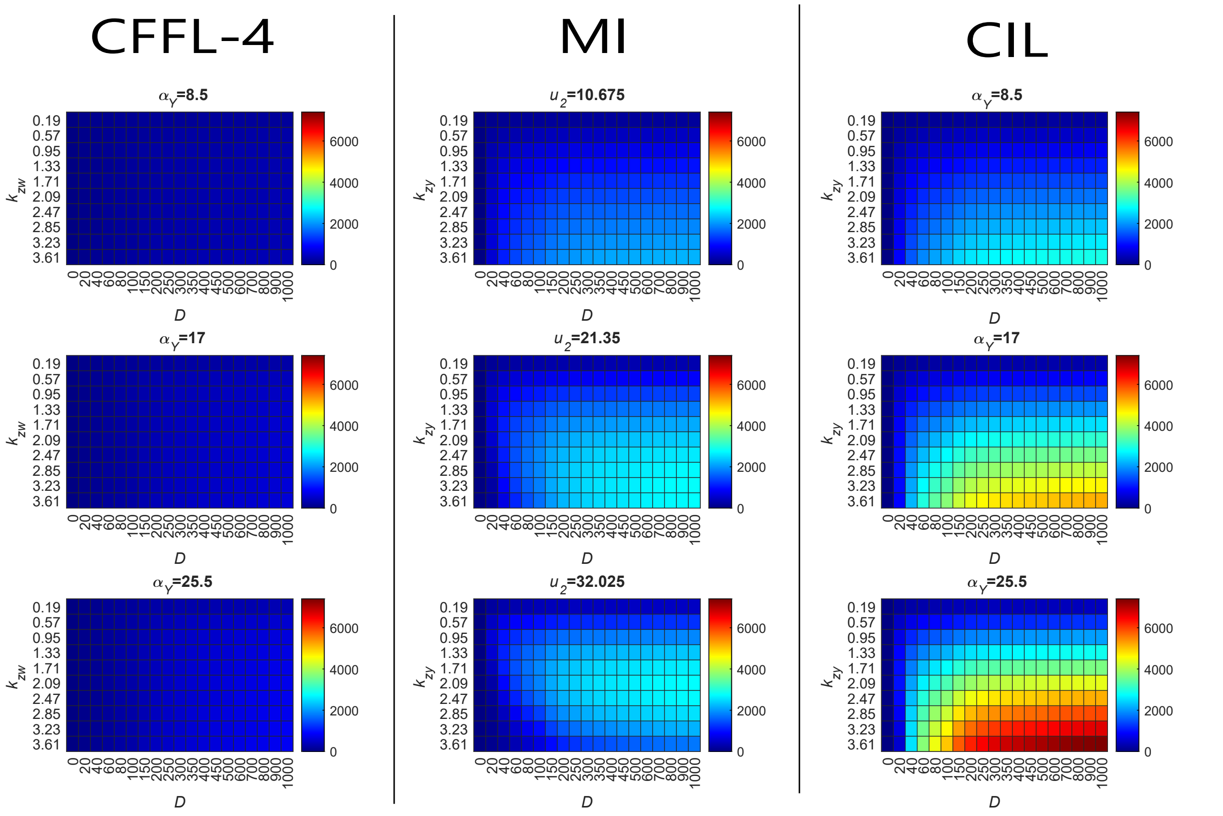

Figure 2: From the left to right, the output response of CFFL4 (7)-(9), MI (15)-(17), and CIL (22)-(24) systems as the doxycycline, α_Y/u_2 and k_{zy} parameters change.

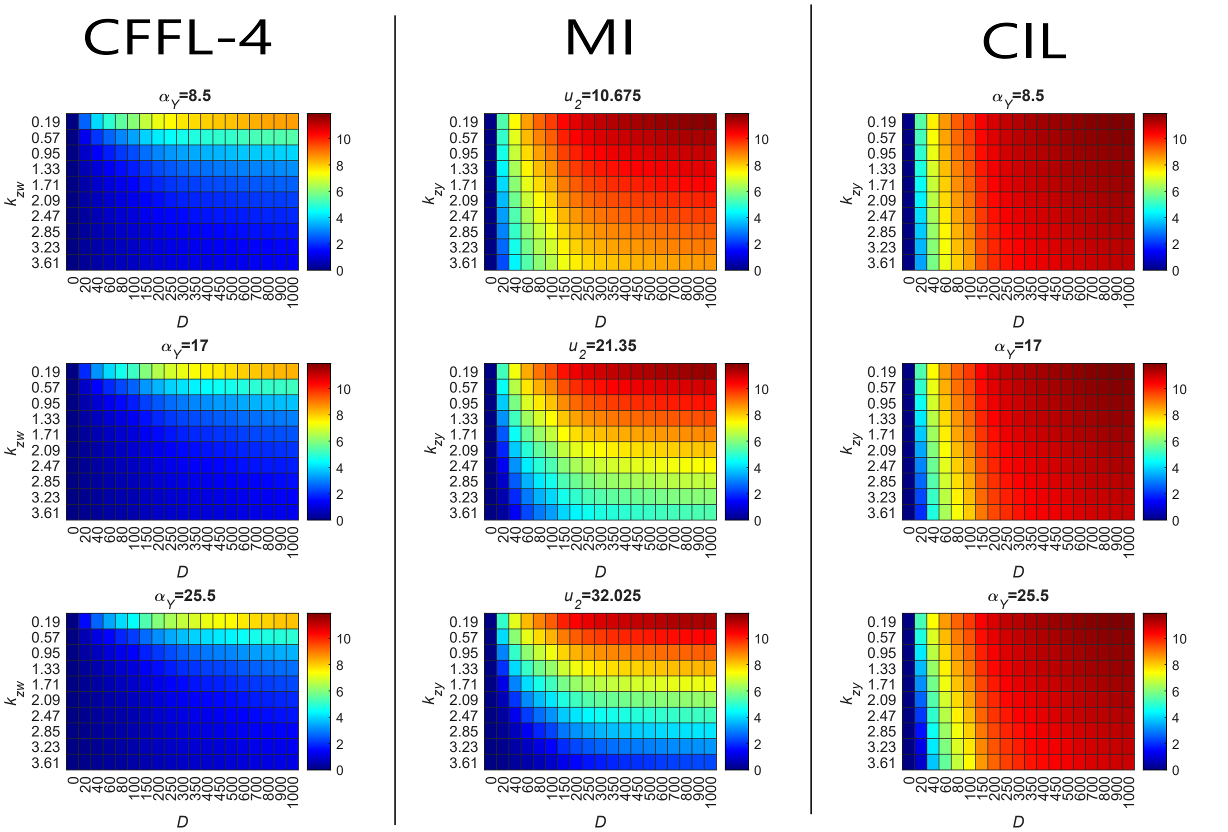

Figure 3: From the left to right, the fold change response of CFFL4 (7)-(9), MI (15)-(17), and CIL (22)-(24) systems as the doxycycline, α_Y/u_2 and k_{zy} parameters change.

APPENDIX A

Derivation of equations for repressor Y

We modeled two distinct mechanisms by which the repressor species Y exerts its action on species Z, according to the specific synthetic circuit we needed to model. In what follows, we assume capital letters to indicate proteins, while the letter m represents mRNA species.

In the case of the CFFL4 model, protein Y cleaves m_Z (i.e. the mRNA of species Z) by transiently binding it, and thus Y can cleave multiple mRNA molecules as described in the following biochemical reactions:

$$\xrightarrow{c_1} \quad m_Z, \tag{39}$$

$$m_Z \quad \xrightarrow{\gamma_{m_Z}} \quad \oslash, \tag{40}$$

$$\xrightarrow{c_2} \quad m_Y, \tag{41}$$

$$m_Y \quad \xrightarrow{\gamma_{m_Y}} \quad \oslash, \tag{42}$$

$$m_Z \quad \xrightarrow{k_Z} \quad Z, \tag{43}$$

$$Z \quad \xrightarrow{\gamma_Z} \quad \oslash, \tag{44}$$

$$m_Y \quad \xrightarrow{k_Y} \quad Y, \tag{45}$$

$$Y \quad \xrightarrow{\gamma_Y} \quad \oslash, \tag{46}$$

$$m_Z + Y \quad \xrightarrow{k_{ZY}} \quad m_Z :: Y, \tag{47}$$

$$m_Z :: Y \quad \xrightarrow{\gamma_{ZY}} \quad \oslash. \tag{48}$$

$$m_Z :: Y \quad \xrightarrow{k_{cut}} \quad Y. \tag{49}$$

The ODEs describing the above reactions are:

$$\frac{dm_Z}{dt} = c_1 - k_{ZY} m_Z Y - \gamma_{m_Z} m_Z, \tag{50}$$

$$\frac{dZ}{dt} = k_Z m_Z - \gamma_Z Z, \tag{51}$$

$$\frac{dm_Y}{dt} = c_2 - \gamma_{m_Y} m_Y, \tag{52}$$

$$\frac{dY}{dt} = k_Y m_Y - k_{ZY} m_Z Y + k_{cut} m_Z :: Y - \gamma_Y Y, \tag{53}$$

$$\frac{dm_Z :: Y}{dt} = k_{ZY} m_Z Y - k_{cut} m_Z :: Y - \gamma_{ZY} m_Z :: Y. \tag{54}$$

Here, we assume that the enzyme is very fast to process the messenger RNA. Therefore, the dynamic (54) is at steady-state and the cleavage action overwhelms the degradation phenomenon of the complex $m_Z :: Z$ (i.e. $k_{cut} \gg \gamma_{ZY}$). Thus the steady-state concentration of the complex:

$$\bar{m}_Z :: \bar{Y} = \frac{k_{ZY} m_Z Y}{k_{cut} + \gamma_{ZY}} \approx \frac{k_{ZY} m_Z Y}{k_{cut}}, \tag{55}$$

is substituted in the equation (53) to make the dynamics of the system (50)-(54) become:

$$\frac{dm_Z}{dt} = c_1 - k_{ZY} m_Z Y - \gamma_{m_Z} m_Z, \tag{56}$$

$$\frac{dZ}{dt} = k_Z m_Z - \gamma_Z Z, \tag{57}$$

$$\frac{dm_Y}{dt} = c_2 - \gamma_{m_Y} m_Y, \tag{58}$$

$$\frac{dY}{dt} = k_Y m_Y - \gamma_Y Y. \tag{59}$$

To further reduce the number of variables, we assume time scale separation between mRNA and protein dynamics, and consider just the equations of proteins where the dynamics of mRNAs are at at steady-state and are equal to:

$$\bar{m}_Z = \frac{c_1}{k_{ZY} Y + \gamma_{m_Z}}, \tag{60}$$

$$\bar{m}_Y = \frac{c_2}{\gamma_{m_Y}}. \tag{61}$$

With these assumptions, we can reduce the model (50):(54) as follows:

$$\frac{dZ}{dt} = \frac{a_1}{1 + k_{zy} Y} - \gamma_Z Z, \tag{62}$$

$$\frac{dY}{dt} = b_1 - \gamma_Y Y, \tag{63}$$

where $k_{zy} = \frac{k_{ZY}}{\gamma_{m_Z}}$, $a_1 = k_Z c_1$, and $b_1 = k_Y \frac{c_2}{\gamma_{m_Y}}$. The reduced model basically entails just a single repression performed by Y on Z at the post-translational level.

When modelling the MI and CIL models, we assumed that protein Y cleaves m_Z (i.e. the mRNA of species Z) by irreversibly binding it, and thus one molecule of Y cleaves only one mRNA molecule in a 1:1 stochiometric fashion. This interaction is described by the following biochemical reaction:

$$\xrightarrow{c_1} \quad m_Z, \tag{64}$$

$$m_Z \quad \xrightarrow{\gamma_{m_Z}} \quad \emptyset, \tag{65}$$

$$\xrightarrow{c_2} \quad m_Y, \tag{66}$$

$$m_Y \quad \xrightarrow{\gamma_{m_Y}} \quad \emptyset, \tag{67}$$

$$m_Z \quad \xrightarrow{k_Z} \quad Z, \tag{68}$$

$$Z \quad \xrightarrow{\gamma_Z} \quad \emptyset, \tag{69}$$

$$m_Y \quad \xrightarrow{k_Y} \quad Y, \tag{70}$$

$$Y \quad \xrightarrow{\gamma_Y} \quad \emptyset, \tag{71}$$

$$m_Z + Y \quad \xrightarrow{k_{ZY}} \quad m_Z :: Y, \tag{72}$$

$$m_Z :: Y \quad \xrightarrow{\gamma_{ZY}} \quad \emptyset. \tag{73}$$

Thus, the dynamics of the involved species can be described by the following model:

$$\frac{dm_Z}{dt} = c_1 - k_{ZY} m_Z Y - \gamma_{m_Z} m_Z, \tag{74}$$

$$\frac{dZ}{dt} = k_Z m_Z - \gamma_Z Z, \tag{75}$$

$$\frac{dm_Y}{dt} = c_2 - \gamma_{m_Y} m_Y, \tag{76}$$

$$\frac{dY}{dt} = k_Y m_Y - k_{ZY} m_Z Y - \gamma_Y Y, \tag{77}$$

$$\frac{dm_Z :: Y}{dt} = k_{ZY} m_Z Y - \gamma_{ZY} m_Z :: Y. \tag{78}$$

Also here we assume that the mRNAs are at steady-state when the others are evolving:

$$\bar{m}_Z = \frac{c_1}{k_{ZY} Y + \gamma_{m_Z}}, \tag{79}$$

$$\bar{m}_Y = \frac{c_2}{\gamma_{m_Y}}. \tag{80}$$

Following this, we can simplify the model (74)-(78) as follow:

$$\frac{dZ}{dt} = \frac{a_1}{1 + k_{zy} Y} - \gamma_Z Z, \tag{81}$$

$$\frac{dY}{dt} = b_1 - kY \frac{a_1}{1 + k_{zy} Y} - \gamma_Y Y, \tag{82}$$

where $k = \frac{k_{ZY}}{k_Z}$. The derived model has both species Y and m_Z characterized by a reduction due to the cleavage process, thus resulting in a mutual repression between Y and Z at post-translational level.

Table 1: Models' parameters

Parameter	Value(s)	Unit	Reference
u_1	19.21	$a.u.\ h^{-1}$	Fitting
u_2	21.35	$a.u.\ h^{-1}$	Fitting
α_Z^0	0.089	$a.u.\ h^{-1}$	Fitting
α_Z	8.91	$a.u.\ h^{-1}$	Fitting
α_W^0	2.40	$a.u.\ h^{-1}$	-
α_W	17.00	$a.u.\ h^{-1}$	-
α_Y^0	2.40	$a.u.\ h^{-1}$	Fitting
α_Y	17.00	$a.u.\ h^{-1}$	Fitting
n_Z	1.00	n.d.	Fitting
k_Z	923.94	a.u.	Fitting
n_W	1.00	n.d.	-
k_W	989.15	a.u.	-
n_Y	1.00	n.d.	Fitting
k_Y	989.15	a.u.	Fitting
γ_X	0.51	h^{-1}	[1]
γ_Y	0.13	h^{-1}	[2]
γ_W	0.13	h^{-1}	[2]
γ_Z	0.74	h^{-1}	[3]
k_{zw}	0.20	a.u.$^{-1}$	-
k_{zy}	0.20	a.u.$^{-1}$	Fitting
k	1.64	a.u.$^{-1}$	Fitting
D	0-1000	$ng\ ml^{-1}$	Experimental setup

References

[1] Elisa Pedone et al. “A tunable dual-input system for on-demand dynamic gene expression regulation”. In: *Nature communications* 10.1 (2019), pp. 1–13.

[2] Matomo Sakari et al. “Effective RNA knockdown using CRISPR-Cas13a and molecular targeting of the EML4-ALK transcript in H3122 lung cancer cells”. In: *International journal of molecular sciences* 21.23 (2020), p. 8904.

[3] Marco Santorelli et al. “Reconstitution of an ultradian oscillator in mammalian cells by a synthetic biology approach”. In: *ACS Synthetic Biology* 7.5 (2018), pp. 1447–1455.

Supplementary Tables

Supplementary Table 1. Plasmids used in this study.

Plasmid name	Figure
pCMV-CasRx(NLS)-T2A-mCherry	2a, 2b, 2c, 2d, 2e, 2f, S1
pCMV-gLuc_pCMV-RedFirefly	2a, 2b, S3
pCMV-gLuc-DR_pCMV-RedFirefly	2a, 2b, S3
pCMV-gLuc-4xDR_pCMV-RedFirefly	2a, 2b, S3
pCMV-rtTA3G	2, 4, S2, S4, S5
pTRE3G-gLuc_pCMV-RedFirefly	2c, 2d, 2e, 2f, 2g, 2h, 2i, 2l
pTRE3G-gLuc-DR_pCMV-RedFirefly	2c, 2d, 2e, 2f, 2g, 2h, 2i, 2l
pCMV/TO-CasRx(NLS)-T2A-mCherry	2g, 2h, 2i, 2l, 3, 4, S3, S4
pMRE-Fluc	3
pMRE-rtTA3G	3
pTRE3G-Fluc	3
pTRE3G-Fluc-DR	3
phRL-TK (Promega)	3
pCMV-HSV-TK (pAL119-TK; Addgene: #21911)	4a, 4b
pTRE3G-HSV-TK-DR	4a, 4b, 4c
AuH1: pCMV-E2A(DBP)-P2A-E4(Orf6)	S8
AuH2: pCMV-E4(Orf6)-P2A-E2A(DBP)	S8
AuH3: pCMV-E2A(DBP)-IRES-E4(Orf6)	4d, 4e, 4f, S8, S9
AuH4: pCMV-E4(Orf6)-IRES-E2A(DBP)	S8
pTRE3G-AuH3	4d,e,f
pTRE3G-AuH3-DR	4d,e,f
pVaRNA-I	4d, 4e, 4f, S9
pHelper (HAdV-5 E2, E4, VaRNA)	4d, 4e, 4f, S8, S9
pPackaging (AAV2 Rep/Cap)	4d, 4e, 4f, S8, S9
pTransgene (pAAV2.1-CMV-EGFP)	4d, 4e, 4f, S8, S9
pCMV/TO-gLuc	S2
pCMV-RedFirefly (Thermofisher)	S2
pEF1a-rtTA3G	S7
pPGK-rtTA3G	S7
